# Age-Related Breakpoints in Pacing Variability and Performance in Masters Swimmers: A Segmented Regression Analysis of World Championship Male and Female Data

**DOI:** 10.3390/jfmk11010078

**Published:** 2026-02-15

**Authors:** Sabrina Demarie, Flavia Guidotti, Christel Galvani, Veronique L. Billat

**Affiliations:** 1 Department of Movement, Human and Health Sciences, University of Rome “Foro Italico”, 00135 Rome, Italy; 2 Department of Human Sciences and Promotion of the Quality of Life, San Raffaele Roma Open, 00166 Rome, Italy; 3 Exercise & Sport Science Laboratory, Università Cattolica del Sacro Cuore, 20123 Milan, Italy; christel.galvani@unicatt.it; 4 Laboratoire Informatique, Bio-Informatique et Systèmes Complexes (IBISC), EA 4526, Université Paris-Saclay, 91034 Evry, France; veroniquelouisebillat@gmail.com

**Keywords:** effort distribution, swimming stroke, competitive swimming, race duration, swimming distance, cadence, aging, performance decline, stroke-specific analysis, sex, gender

## Abstract

**Background:** Pacing critically influences swimming performance. In master swimmers, aging leads to performance decline, but the age at which pacing becomes unstable, and whether this precedes performance loss, remains unclear. **Objective:** This cross-sectional retrospective study analyzed sex, distance and stroke-specific age-related breakpoints in pacing variability (CV) and performance (RT) in master swimmers. **Methods:** A total of 13,822 swimmers (7417 men and 6405 women; age 25–99 years) competing at the World Aquatics Masters Championships (2023–2025) were included. **Results:** CV showed the strongest association with RT (r = 0.173, *p* < 0.001). Overall, CV worsened significantly earlier (52 years, +2.82%/year) than RT (82 years, +0.51%/year; *p* < 0.001). In women, CV deterioration began at ~50 years, while RT was maintained until ~85 years; this was particularly pronounced in short-distance events (pacing breakpoint at 35 years). Men displayed more synchronized decline patterns. Age breakpoints of CV and RT were coincident in freestyle and breaststroke (82 years). Backstroke and butterfly demonstrated RT breakpoints at 47 and 67 years, respectively, with CV occurring at 72 years. **Conclusions:** These findings indicate that CV generally deteriorates years before RT and represents a stroke, sex and distance-specific marker of accelerated functional decline in elite master swimmers. Monitoring CV may provide early warning of impending performance deterioration informing timely, targeted training interventions to extend athletic longevity.

## 1. Introduction

### 1.1. Pacing and Performance in Cyclic Locomotor Activities

As in running and cycling, pacing and its variability are crucial determinants of performance in swimming [1,2,3,4,5,6,7,8,9]. However, despite being cyclic locomotor activities, they elicit distinct metabolic and physiological responses due to differences in body position, muscle recruitment patterns, environmental conditions, and biomechanical efficiency [10]. Swimming, conducted in a horizontal, buoyant environment, engages a larger proportion of upper-body musculature and imposes unique respiratory challenges due to hydrostatic pressure on the chest wall increasing the work of breathing [11,12]. Maximal oxygen uptake values are typically 10–15% lower during swimming compared to running or cycling, and thermoregulatory demands differ markedly [13]. These modality-specific physiological constraints shape different training adaptations, pacing strategies, and performance outcomes across disciplines.

### 1.2. Aging and Performance Decline in Masters Sports

Research in masters sports shows that performance declines and disruptions in pacing tend to accelerate exponentially after ages 70–75, with considerable individual variation shaped by factors such as training history, sex, genetics, and injury background [14,15,16,17,18,19]. However, this decline is not inevitable or irreversible [20]. A landmark case study by Billat et al. (2017) [21] documented centenarian cyclist Robert Marchand, who, between the ages of 100 and 103, engaged in two years of cadence-focused training that led to a 13% increase in maximal aerobic power and a new world record. This remarkable outcome highlights that neuromuscular and cardiorespiratory systems retain significant plasticity even in extreme old age, particularly when training targets movement frequency and efficiency [21]. By extension, stroke-specific or skill-oriented cadence training may help aging swimmers preserve, or even regain, optimal stroke rate, consistent pacing, and competitive performance. However, the timing, extent, and trainability of such adaptations in the oldest swimmers, and across different swimming strokes, remain inadequately understood [22,23].

### 1.3. Pacing Instability as an Early Marker of Functional Decline

On these premises, it can be hypothesized that identifiable age-related breakpoints in pacing stability exist among competitive master swimmers, and that the onset of pacing instability may serve as an early physiological marker of functional decline. Clarifying these patterns, along with the underlying physiological and biomechanical constraints, could empower coaches to tailor training and race strategies more effectively for older athletes [14,15,16]. Identifying age breakpoints for pacing instability may provide an early indicator of functional decline, enabling timely training interventions to sustain competitive performance and extend athletic longevity among aging swimmers.

### 1.4. Study Objectives

The aim of this study was to identify distinct age-associated breakpoints that mark the onset of increased pacing variability and performance deterioration in elite master swimmers, using segmented regression analysis of international competition data, and to characterize stroke-specific patterns of age-related decline that may inform targeted training interventions.

## 2. Materials and Methods

### 2.1. Study Design and Data Collection

This study employed a retrospective observational cross-sectional design analyzing competition data from World Aquatics Masters Championships held in three locations: Kyushu, Japan (2023), Doha (2024), and Singapura, Singapura (2025). The validity of using split times from official competitions as a reliable measure of pacing behavior has been previously reviewed, confirming that split times recorded during official World Aquatics events are highly reliable due to standardized electronic timing systems (±0.01 s accuracy) [5].

### 2.2. Participants and Sociodemographic Characteristics

Performance data were retrieved from the World Aquatics official website (https://www.worldaquatics.com), accessed 30 September 2025. All timing data was taken using Omega automatic officiating equipment (Quantum Timer with OSB11 touchpads, version SW1.6.10, Swiss Timing L.T.D., Corgémont, Switzerland) certified by World Aquatics.

The dataset comprised 13,822 individual performances (7417 male, 6405 female) by swimmers from 85 different countries across six continents: Africa (*n* = 452), Asia (*n* = 8836), Europe (*n* = 3066), North America (*n* = 1461), South America (*n* = 662), and Oceania (*n* = 463). Swimmers ranged in age from 25 to 99 years, capturing the full spectrum of standard masters swimming categories regulated by World Aquatics. The analysis included race distances spanning 100 m to 800 m across all four competitive swimming strokes: freestyle, backstroke, breaststroke, and butterfly.

The wide age range (25–99 years) was intentionally retained to identify critical transition periods throughout the aging process and to capture breakpoints across the full lifespan trajectory of master swimmers [24,25,26]. This comprehensive age range allowed for the detection of early markers of decline in younger masters athletes and the characterization of extreme aging patterns in centenarian and near-centenarian swimmers, thereby providing a complete picture of age-related performance dynamics that would be obscured by age stratification or exclusion of extreme ages.

### 2.3. Data Collection and Ethical Considerations

All data were collected anonymously; each participant was assigned an alphanumeric code identifying sex, age, competition, and ranking position. Apart from sex and age, no other personal data were recorded. Consent to publish competition data was given mandatorily at the time the swimmers’ registration, and as their owners did not restrict their use, all data displayed in the World Aquatics database are freely and publicly available (https://www.worldaquatics.com/privacy-policy; accessed on 23 January 2026).

The study used only publicly available, anonymized competition data where participant identities were protected by alphanumeric codes. In accordance with the Declaration of Helsinki guidelines for observational research using publicly available anonymized data, formal ethical approval was not required. This study did not constitute a clinical trial and did not require registration under clinical trial databases (e.g., ClinicalTrials.gov or national equivalents), as it involved no experimental intervention and no identifiable human subjects, but only secondary analysis of de-identified competition records already in the public domain [26].

### 2.4. Study Flowchart

**Figure 1:**
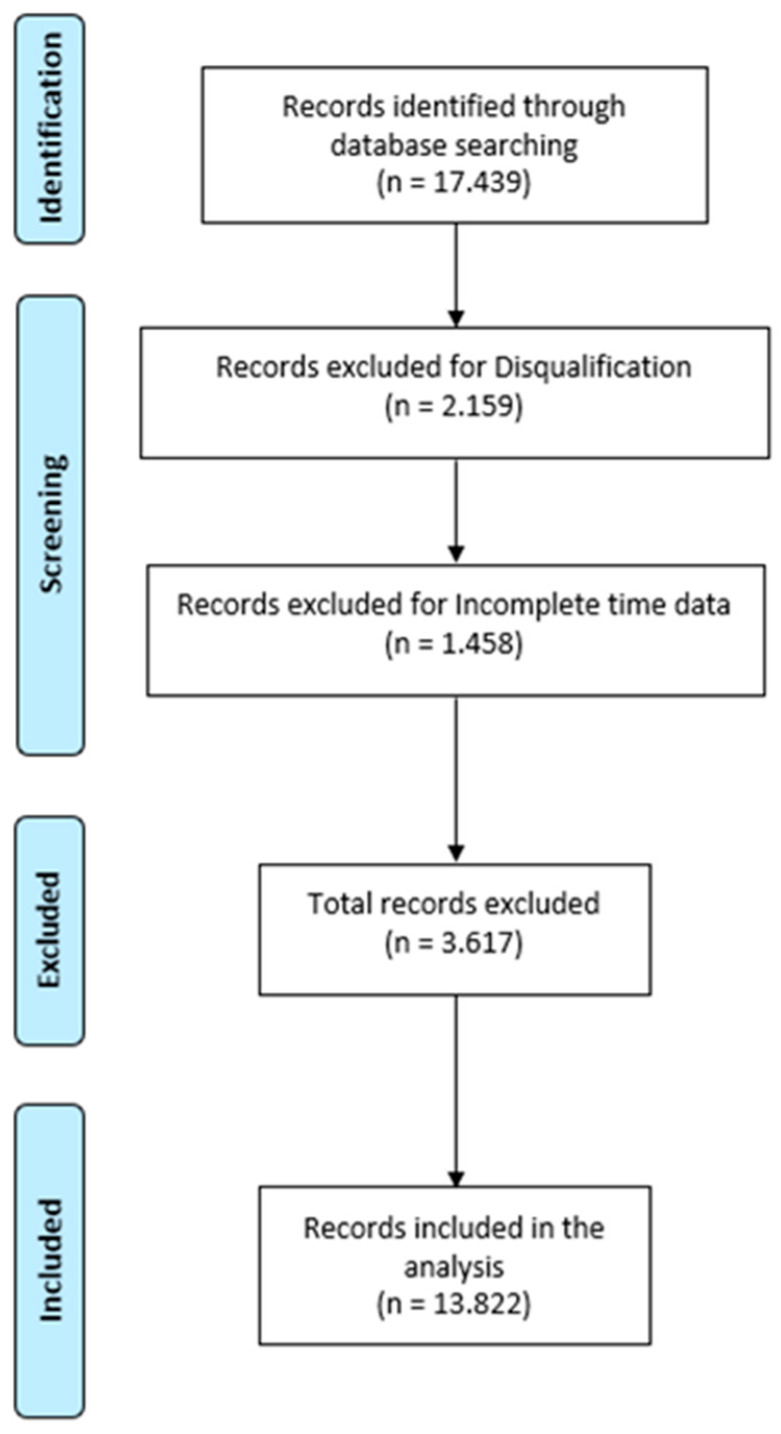
Flow diagram of the data selection process.

The flowchart in Figure 1 depicts the data screening process, the inclusion/exclusion criteria used to identify the eligible performances (*n* = 13,822) from the three championships, and the final sample composition.

### 2.5. Inclusion and Exclusion Criteria

Inclusion criteria:

(1) Official finisher in individual swimming events at the specified championships;
(2) Complete split-time data available;
(3) Adherence to World Aquatics competition rules during the event;
(4) Participants in individual events;
(5) Participants in single stroke events.

Exclusion criteria

(1) Disqualification by officials for rule violations (including false starts, stroke infractions, or improper turns);
(2) Incomplete or missing split-time data for any race segment.

These criteria ensured that only valid, complete performance records with reliable timing data were included in the analysis.

### 2.6. Study Timeline and Protocol

**Figure 2:**
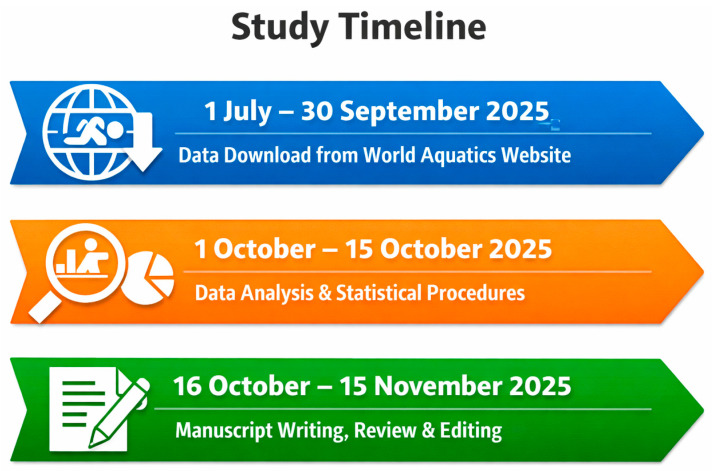
Overview of the study timeline outlining the three main phases.

This retrospective analysis examined the competition results from three consecutive World Aquatics Masters Championships (2023–2025). Data extraction and analysis were conducted between July and September 2025, as represented in Figure 2. The analysis timeline is presented in the flowchart, showing the data collection periods for each championship and corresponding analysis phases.

### 2.7. Variables Analyzed

Race Time %WR (%): time to complete the race, expressed as a percentage of world record. It represents how close the swimmer’ race time is to the best possible time (the world record) for the corresponding year of the competition, sex and age. A value of 100% means the swimmer performed exactly as fast as the world record holder for matching age and sex. The higher the percentage, the slower the performance compared to the world record [27].

Pacing variability (CV, %): coefficient of variation calculated as the standard deviation divided by the mean of all within-race split durations (every 50 m for 100–400 m races and every 100 m for 800 m races), multiplied by 100.

Split time 1 half (%): mean split time for the first half of the race, expressed as a percentage of the mean split time of the entire race. Values lower than 100% indicate that the first half was faster than the race average.

Split time 2 half (%): mean split time for the second half of the race, expressed as a percentage of the mean split time of the entire race. Values greater than 100% indicate that the second half was slower than the race average.

Delta 1–2 half (%): difference between the first and second halves of the race, calculated as Split time 1 half minus Split time 2 half. Negative values indicate that the second half of the race is slower than the first half (positive split), whereas positive values indicate a faster second half (negative split).

### 2.8. Sample Size Estimation

While no a priori sample size estimation was performed due to the observational and retrospective nature of the study using existing competition data, we conducted post hoc power analysis using G*Power 3.1, demonstrating a power > 0.99 to detect small effects (f = 0.10) with α = 0.05 and β = 0.20. Moreover, the final dataset comprised 13,822 performances (7417 male and 6405 female), distributed across multiple age groups, strokes and race distances. This large sample provides substantial statistical power for detecting small associations between pacing indices and performance and for estimating segmented regression models with reasonable precision. The results should therefore be interpreted in terms of effect size and practical relevance, rather than statistical significance alone.

### 2.9. Data Analysis Strategy

Descriptive Statistics: Descriptive statistics were calculated for all variables and presented as mean ± standard deviation (SD).

Normality Assessment: Normality of data distribution was verified using the Shapiro–Wilk test. Given the large sample size, a visual inspection of Q-Q plots and histograms was also conducted to assess distributional characteristics.

Correlation Analysis: Pearson product–moment correlation coefficients (r) were computed between Race Time %WR and each pacing-related variable (CV, Split time 1 half, Split time 2 half, and Δ2–1 half). Given the large sample size, correlations were interpreted primarily according to their magnitude (effect size) rather than *p*-values alone, using the following guidelines: |r| < 0.10 = negligible; 0.10–0.39 = small; 0.40–0.69 = moderate; and ≥0.70 = large.

Effect Size Calculation: Cohen’s d was calculated by dividing the difference between group means by the pooled standard deviation to quantify the magnitude of group differences. Effect sizes interpretation: |r|: <0.10 = negligible; 0.10–0.39 = small; ≥0.40–0.79 = moderate; and ≥0.80 = large. Effect Size Interpretation: f^2^: ≥0.02 = small; ≥0.15 = medium; and ≥0.35 large.

Segmented Regression Analysis: To identify the age at which race performance and pacing variability changed in their rate of deterioration, piecewise (segmented) linear regression models with a single breakpoint were fitted to mean values of Race Time %WR and CV aggregated by age group. Bootstrapping with 1000 iterations was used to obtain 95% confidence intervals for breakpoint estimates. After identifying the breakpoint age, log-linear segmented regressions allowing different slopes before and after the breakpoint were fitted to quantify the annual rate of change in each variable [28,29].

Composite Risk Score: To summarize stroke-specific vulnerability to accelerated age-related decline, a composite risk score was derived from the segmented models. For each stroke, three parameters were extracted: (i) age at the breakpoint in Race Time %WR (earlier breakpoint = higher risk); (ii) absolute annual rate of change in Race Time %WR after the breakpoint (faster post-breakpoint deterioration = higher risk); and (iii) goodness of fit of the segmented model (R^2^; lower R^2^ = higher uncertainty and therefore higher risk). Each parameter was standardized to a 0–1 scale across the four strokes by min–max rescaling, yielding three sub-scores: Age Risk, Acceleration Risk, and Reliability Risk. The composite risk score (0–100) was computed as a weighted sum of these sub-scores: composite score = (Age Risk × 0.40) + (Acceleration Risk × 0.40) + (Reliability Risk × 0.20). Scores were interpreted descriptively as follows: 0–24 = low decline risk; 25–49 = moderate risk; 50–74 = high risk; and ≥75 = very high risk. This framework was intended as a comparative descriptive tool across strokes and as an exploratory aid for identifying intervention opportunities, not as an individual predictive model [30].

Statistical Software: Data analysis was conducted using Python version 3.15 (Python Software Foundation, Beaverton, OR, USA) and IBM SPSS Statistics version 30 (IBM Corp., Armonk, NY, USA), with statistical significance set at *p* < 0.05.

## 3. Results

### 3.1. Participant Characteristics

Table 1 and Table 2 present the distribution of participants by age group and sex. The sample was relatively well distributed across age groups, with larger numbers of participants in the 50–69 age range. The largest age groups were 55–59 years (*n* = 1469) and 60–64 years (*n* = 1517), while the smallest groups comprised the oldest competitors (95–99 years, *n* = 5). Sex distribution was relatively balanced, with 53.6% male and 46.4% female participants.

### 3.2. Associations Between Pacing Variables and Race Time

Table 3 summarizes the correlations between pacing-related indices and Race Time %WR. All associations were statistically significant (*p* < 0.001) but small in magnitude (|r| ≤ 0.18; f^2^ = 0.03).

Pacing variability (CV) showed the largest positive correlation with Race Time %WR (r = 0.173, *p* < 0.001, 95% CI: 0.159–0.188), indicating that swimmers with more variable pacing tended to perform at higher Race Time %WR. The Cohen’s d effect size for this association was 0.347 (small).

Split time 1 half was negatively correlated with Race Time %WR (r = −0.147, *p* < 0.001, 95% CI: −0.162–−0.133), suggesting that performances characterized by an excessively fast first half (i.e., very low relative Split time 1 half) tended to be associated with slower overall race times, consistent with a “fast-start” penalization.

Split time 2 half was positively correlated with Race Time %WR (r = 0.130, *p* < 0.001, 95% CI: 0.115–0.144), meaning that relatively slower second-half splits were associated with poorer race performance.

Δ2–1 half also exhibited a small negative correlation with Race Time %WR (r = −0.147, *p* < 0.001, 95% CI: −0.162–−0.133). Given that a more negative Δ2–1 value indicates second-half slowing relative to first-half speed, this pattern suggests that greater end-race slowing (positive split) is related to worse overall performance.

Taken together, these findings indicate that more stable and balanced pacing, reflected by lower CV and smaller differences between the first and second halves, is associated with better race outcomes in master swimmers across all ages and strokes.

### 3.3. Age Breakpoint Analysis: Pooled Data Across All Strokes and Sexes

Segmented regression analysis revealed distinct age-related breakpoints for pacing variability and race performance. Table 4 presents the key findings from the segmented models fitted to pooled data across all four strokes and both sexes.

The analysis identified two distinct age breakpoints:

1. Pacing Variability Breakpoint (52 years): Prior to age 52, pacing variability increased at a relatively modest rate (+0.18%/year). After the breakpoint, the rate of deterioration accelerated dramatically to +2.82%/year, a 15.7-fold increase. The high R^2^ value before the breakpoint (0.764) indicates a reliable pre-breakpoint relationship (f^2^ = 1.46).
2. Race Performance Breakpoint (82 years): Race Time %WR showed minimal change before age 82 (+0.28%/year) but then deteriorated significantly thereafter (+0.51%/year), displaying a 1.82-fold acceleration (f^2^ = 12.34). This breakpoint occurs 30 years after the pacing breakpoint.
3. Temporal Dissociation: The most striking finding is the 30-year temporal separation between pacing and performance breakpoints. Pacing instability emerges decades before measurable performance deterioration becomes pronounced, suggesting that pacing variability is a sensitive early warning indicator of age-related decline.

### 3.4. Age Breakpoint Analysis by Sexes

**Figure 3:**
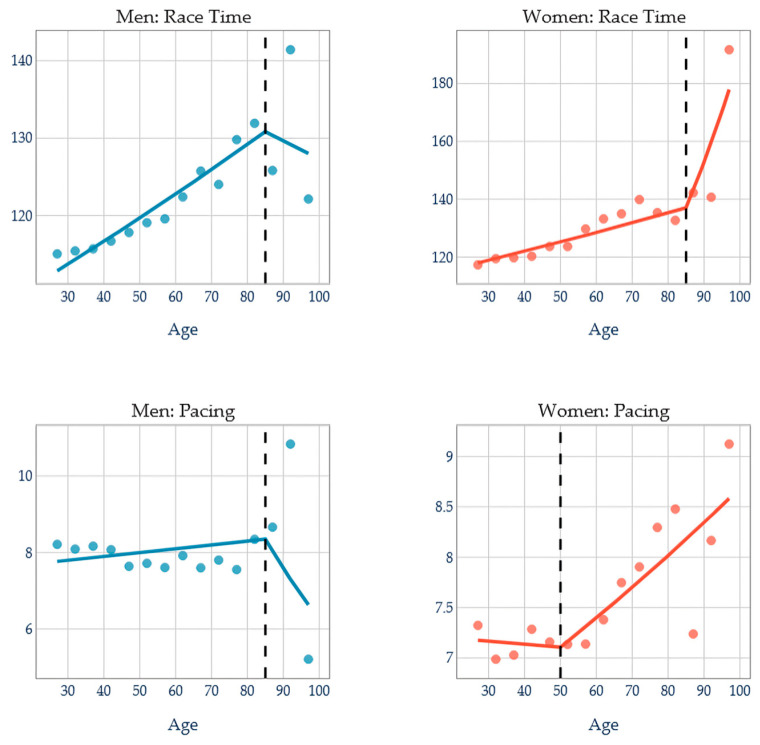
Breakpoint age analysis by sex.

As depicted in Figure 3, women’s Race Times present a significant slope change (*p* = 0.001) at the breakpoint, with a large effect size (f^2^ = 6.22), portraying an 8.4× acceleration in deterioration after age 85 years. Women’s pacing approaches significance (*p* = 0.082) and presents a moderate effect size (f^2^ = 2.13) with a breakpoint age at 50 years representing a meaningful change in pacing trajectory. Neither the Race Time nor pacing men’s models show a significant slope change at the breakpoint, with a low effect size (f^2^ = 0.19) representing high individual variability in men’s aging patterns.

**Figure 4:**
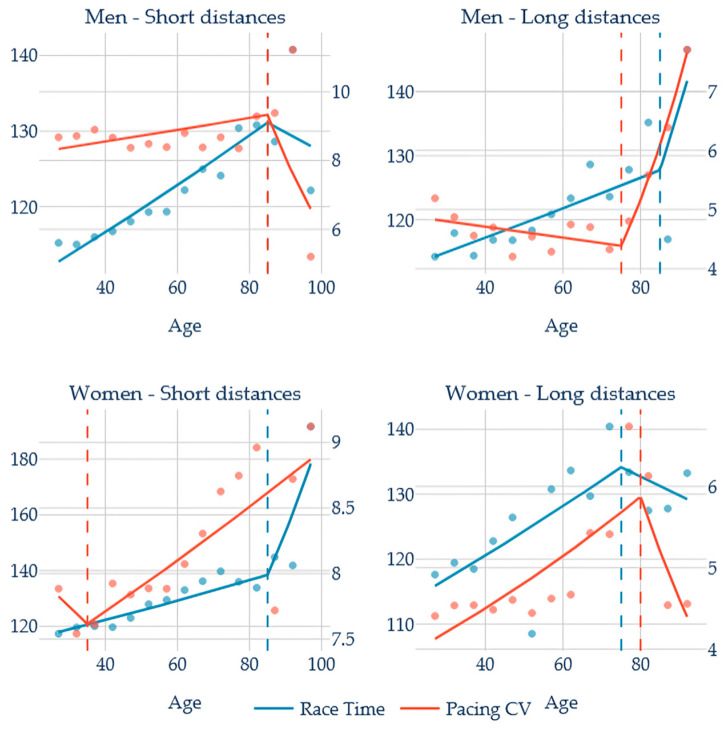
Breakpoint age analysis of sex and distance interactions; Short distances: 100 m and 200 m; Long distances: 200 m and 400 m.

Figure 4 shows an extremely early pacing breakpoint (−50 years) for women’s short distances and a reversed pattern for long distances (+5 years). Men pacing and race time show the same breakpoints in the short distances, and the pacing breakpoint 10 years earlier at long distances.

### 3.5. Stroke-Specific Age Breakpoints and Performance Trajectories

**Figure 5:**
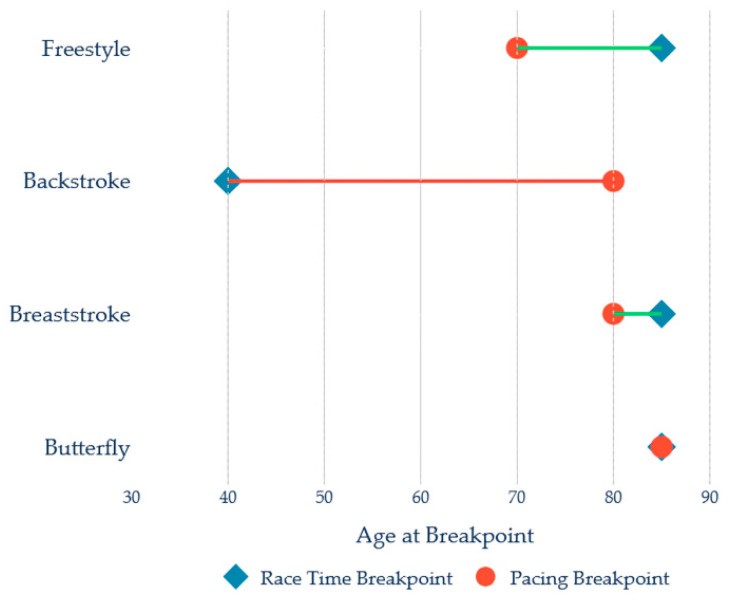
Breakpoint age analysis for the four swimming strokes. Lines: Green = Pacing declines first (typical pattern); Red = Race time declines first.

Figure 5 and Table 5 illustrate stroke-specific age breakpoints for both race performance and pacing variability. Marked heterogeneity emerged across the four swimming strokes.

Stroke-specific patterns analysis revealed that:

1. Freestyle: Both pacing and performance breakpoints coincided at 82 years. However, freestyle displayed the steepest post-breakpoint slope for performance (6.58%/year), indicating the most rapid deterioration once decline began. Pacing also deteriorated steeply post breakpoint (3.15%/year). This pattern suggests that freestyle performers maintain stability until very advanced ages, after which decline accelerates sharply.
2. Breaststroke: Like freestyle, breaststroke showed coincident breakpoints at 82 years for both pacing and performance. However, the post-breakpoint rise in Race Time %WR was more gradual (1.02%/year), resulting in a slower rate of performance decline compared to freestyle. This more gradual deterioration suggests greater resilience in breaststroke performance with advancing age.
3. Backstroke: Backstroke displayed the earliest performance breakpoint of all strokes (47 years), yet the smallest post-breakpoint slope in Race Time %WR (0.30%/year). Notably, pacing variability remained relatively stable until approximately 72 years, representing a 25-year lag behind performance decline. This dissociation suggests that backstroke performance begins to decline in the late 40s through mechanisms not directly reflected in pacing variability, possibly related to stroke-specific biomechanical constraints (e.g., neck extension, shoulder range of motion).
4. Butterfly: Butterfly combined an earlier breakpoint in pacing variability (67 years) with a subsequent breakpoint in race performance (72 years), revealing a 5-year advance in pacing deterioration. The post-breakpoint slope for race performance (2.07%/year) and pacing variability (3.52%/year) were both substantial, indicating that butterfly swimmers experience noticeable deterioration in both pacing control and race times once decline begins.

Stroke age breakpoint analysis shows that all strokes, except breaststroke pacing, show very good model fits (R^2^ > 0.74) with large effect sizes (f^2^ > 0.35). Regression coefficients were always statistically significant (*p* < 0.05) and had large effect sizes (f^2^) ranging from 0.41 for breaststroke pacing to 22.05 for backstroke pacing.

Notable variations include freestyle showing the steepest post-breakpoint performance decline; breaststroke showing late coincident breakpoints but gradual deterioration; backstroke showing the earliest performance breakpoint but the slowest subsequent decline; and butterfly showing early pacing deterioration (5 years before performance).

### 3.6. Stroke-Specific Critical Age Windows and Risk Profiles

Based on stroke-specific segmented regression results, a composite risk score was calculated to integrate the timing (breakpoint age), magnitude (post-breakpoint slope), and reliability (R^2^) of age-related decline for each stroke.

The following stroke-specific patterns, illustrated in Table 6, are consolidated into critical age windows for enhanced monitoring and targeted intervention:

1. Backstroke (40–49 years): Moderate risk driven by earliest performance onset.
2. Butterfly (60–79 years): Moderate risk with early pacing deterioration (67 years).
3. Freestyle and Breaststroke (80–89 years): Moderate and low-moderate risk, respectively, with late but potentially rapid decline in freestyle.

Figure 3 provides a visual representation of these stroke-specific patterns.

### 3.7. Relationship Between Pacing Breakpoint and Performance Breakpoint Timing

An additional observation emerged: strokes with earlier performance breakpoints (backstroke, 47 years) tend to have slower post-breakpoint performance slopes, whereas strokes with later breakpoints (freestyle, 82 years) tend to have steeper post-breakpoint slopes. This suggests an inverse relationship between breakpoint timing and decline velocity; strokes that decline earlier may do so more gradually, while strokes that remain stable for longer may experience more abrupt deterioration once decline begins. This relationship has practical implications: backstroke swimmers should begin preventive strategies in their 40s, whereas freestyle swimmers can maintain intensive training longer but may need rapid adaptation strategies once decline begins in their 80s.

## 4. Discussion

### 4.1. Main Findings and Significance

The main findings of this study are that, in master swimmers competing at recent World Aquatics Masters Championships, pacing variability exhibits an age-related breakpoint several decades earlier than overall race performance, and that both the timing and magnitude of subsequent deterioration are stroke-specific [4,5]. When all strokes were pooled, pacing variability began to increase markedly from the early 50s (breakpoint at 52 years, +2.82%/year thereafter), whereas a breakpoint in Race Time %WR was only observed at approximately 80 years of age (+0.51%/year thereafter). This 30-year temporal separation suggests that a loss of pacing stability is an early marker of age-related deterioration in competitive swimming, preceding the point at which race times start to diverge more rapidly from age and sex group world records.

These results demonstrate that pacing control is a sensitive indicator of aging-related changes in neuromuscular and metabolic regulation, potentially deteriorating before more global measures of performance capacity. This finding aligns with research in human longevity showing that early detection of functional markers can facilitate timely intervention before catastrophic decline. In human populations, the identification and characterization of successful agers who maintain a high physiological reserve in response to stress at an advanced age, along with physiological functions that are significantly superior to those of their age group, represent a promising strategy to assess and investigate health and disease over the life course [31,32,33].

### 4.2. Physiological and Metabolic Characteristics of Swimming

Swimming performance is influenced by several key energetic factors, including peak post-exercise blood lactate concentration ([La^−^]peak), the swimming velocity at a blood lactate concentration of 4 mmol·L^−1^ (sLT), and maximal oxygen uptake (VO_2_max) [34]. In general, both lactate production and clearance during and after exercise are lower in adults compared to younger individuals [35]. Aging also leads to changes in body composition that affect muscle structure, thereby diminishing the capacity for strength- and power-based activities [36]. A primary contributor to the age-related decline in endurance performance is the progressive reduction in VO_2_max, followed by a decrease in lactate threshold (LT), the exercise intensity at which blood lactate levels rise markedly [24]. In healthy, sedentary adults of both sexes, VO_2_max typically declines by about 10% per decade after the ages of 25–30 [37]. Additionally, muscle strength and power inevitably diminish with advancing age [13]. Collectively, these changes point to an overall reduction in both maximal aerobic and anaerobic power and capacity as individuals grow older [14,16]. Consequently, regular swimming training may play a crucial role in counteracting this decline, helping to preserve or even enhance athletic performance while supporting metabolic health.

### 4.3. Aging, Pacing Control, and Endurance Performance

These results align with previous work in endurance sports showing that aging affects not only maximal physiological capacity but also the ability to regulate effort and maintain stable pacing. Biological studies indicate that it is improbable for a single biomarker to be adequate, considering the complex multisystem characteristics of the aging process, which involves alterations at both molecular and organ-based levels, thereby highlighting the value of a composite score for biological aging [31,38,39]. Studies in pool swimming, open-water events, running and cycling have reported that older athletes often adopt more variable or conservative pacing strategies, even when mean speed is relatively well preserved, likely reflecting changes in neuromuscular function, perception of effort and decision-making under fatigue [2,40,41].

Currently, the molecular mechanisms underlying aging remain inadequately understood, making pharmacological interventions that specifically target aging difficult to achieve. The molecular biomarkers associated with aging show considerable variability, with only a limited number of validation attempts conducted in human subjects. In contrast, physiological biomarkers of aging, which focus on functional, anthropometric, and morphological characteristics, are well-established in extensive human populations and possess a very high predictive value for disease risks, frailty, morbidity, and mortality [39,42]. Lifestyle, behavioral, and environmental factors significantly influence human health and mortality, while many pharmacological and interventional strategies identified in preclinical studies are still pending translation to human applications. These factors, which have demonstrated benefits, should be actively encouraged and promoted at both individual and societal levels [39,42].

Our data extend this concept to elite master swimmers across four strokes, highlighting that the pace-control component of performance is sensitive to aging and may deteriorate before more global indicators such as race time relative to world records [23,43].

### 4.4. Biomechanical and Physiological Mechanisms Underlying Age-Related Decline

Aging significantly influences the biomechanical and technical demands of swimming due to physiological, neuromuscular, and structural changes that occur over time [44]. The age-related stiffening of connective tissues, the joint degeneration, and the loss of skeletal muscle mass lead to decreased stroke power and speed [45,46,47,48]. The lower recovery rate and higher susceptibility to overuse injuries due to tendon stiffness and reduced tissue resilience lead to altered stroke mechanics to avoid pain, which can reinforce inefficiencies [49]. Slower neural processing and reduced motor unit recruitment affect timing and coordination [20,50]. The biomechanical consequence is less synchronized movements between arms, legs, and breathing that can disrupt rhythm and increase energy cost [18].

With aging, stroke length is compromised by the limited shoulder extension during the reach phase and by the reduced force production during the pull phases [49]. Reduced ankle dorsiflexion and hip mobility compromise kick efficiency, especially in flutter and butterfly kicks [51]. Older swimmers often focus on technique refinement, maximizing propulsion per stroke through better hand and forearm pitch, catch mechanics, shorter but more precise pulls, and body alignment [35].

Increased fat mass and decreased lean mass with aging alter buoyancy and the drag profile. Even though higher body fat may improve natural buoyancy (beneficial for horizontal alignment), increased frontal surface area or poor core tone can increase drag. Adding to this, the age-related decline in vestibular function and proprioception affects spatial awareness, leading to difficulty maintaining optimal body roll, head position, and alignment, thus increasing drag [16,52]. Moreover, maximal oxygen uptake (VO_2_max) declines ~1% per year after age 30, together with a lung elasticity decrease [53]. The reduced aerobic capacity limits sustained high-intensity efforts, influencing race strategy and training intensity and++ leading to the adoption of more efficient stroke techniques to conserve energy and maintain pace with less oxygen demand [49,54].

### 4.5. Sex-Specific Aging Patterns

In women participants, the 30-year gap between breakpoints, where pacing control start declining at 50–54 while race time holds until 85, represents the period where race time is still stable but pacing has already started to decline. It could be thus argued that women may need more pacing-specific training around the age of 50 years, thus prioritizing race strategy and split training. Men’s race time and pacing metrics decline together at 85, showing no gap. This suggests that hormonal and physiological differences may affect pacing control earlier in women.

Menopause (age ~50) may play a role. Hormonal changes, in particular estrogen decline, could impact motor control and fatigue perception [55,56]. Testosterone levels, which are significantly higher in men, contribute to greater muscle mass and strength, impacting performance differences between sexes. This hormonal difference is a key factor in the observed performance disparities in athletics, with men generally outperforming women in strength and endurance events [57]. Age-related hormonal changes, such as decreased testosterone in men and estrogen in women, affect muscle mass, bone density, neuromuscular control and perceived exertion influencing performance in master athletes. However, gender hormones seem to poorly influence aging performance decay; therefore, other fundamental bioregulators, or physiological factors, could be relevant [58]. Echocardiographic studies show that male master athletes have a higher left ventricular mass compared to females, attributed to greater end-diastolic dimensions, while women exhibit higher left ventricular systolic global longitudinal strain, indicating differences in cardiac function between sexes [59]. The findings of a study on a group of 146 male and 82 female master athletes revealed that, over the age of 40 years, all metabolic parameters, regarding body composition, blood chemistry, blood pressure, VO_2_max, muscle strength, bone density, and performance, decreased at significantly different rates between sexes. Additionally, bone density exhibited a decline as age progressed that was significant in females only [60].

It has been previously reported that the age-related rates of decline are greater in women than in men only in short-duration events [14]. The disparity in performance between sexes within the age range 19–99 years decreases steadily as the distance increases from 50 m (19 ± 1%) to 1500 m (11 ± 1%). The decline in swimming performance with age at both 50 m and 1500 m is significantly more evident in women than in men. For women, the percentage decline in swimming performance after reaching the age of 50, moving from the age group of 19–24 years to that of 69–74 years, increases progressively from 50 m to 1500 m; conversely, in men, no notable differences were observed in the degree of performance decline with age across the five longest distance events [61].

Accordingly, our analysis revealed that in women, the ability to regulate pacing in short-duration, high-intensity efforts deteriorates substantially earlier than performance outcomes. Short sprint events require fine neuromuscular coordination, rapid force production, and precise distribution of effort over very brief time frames. The early decline in pacing control may therefore reflect an accelerated age-related reduction in neuromuscular precision and central motor regulation, which precedes the observable decline in final race time [62]. A reversed pattern emerged in women competing over long distances, where the pacing breakpoint (80 years) occurred later than the race time breakpoint (75 years), resulting in a positive gap of approximately +5 years. This finding indicates that women retain the capacity to regulate pace effectively even after measurable declines in performance have begun. Such preservation of pacing control in long-distance events may be attributed to sustained aerobic efficiency, metabolic regulation, and experiential or cognitive components of pacing that are less dependent on rapid neuromuscular responses [63,64].

In contrast, men exhibited more homogeneous and delayed breakpoint patterns across distance categories, indicating a similar lifespan trajectory of race time and pacing variability decline regardless of event duration. However, pacing breakpoints differed by distance: pacing control declined earlier in long-distance events (75 years) compared with short-distance events (85 years). This suggests that, in men, the regulation of effort over prolonged durations is more sensitive to aging than the pacing demands of short events [16,33,43].

### 4.6. Stroke-Specific Aging Patterns

Stroke-specific analyses provide additional insight into how aging interacts with the biomechanical and technical demands of different swimming styles.

Freestyle: The coincident pacing and performance breakpoints at 82 years, combined with the steepest post-breakpoint performance slope (6.58%/year), suggest that freestyle swimmers benefit from stroke mechanics that allow relatively stable performance maintenance until very advanced ages. However, once decline begins, it accelerates rapidly. This pattern may reflect the mechanical efficiency of freestyle, the most economical stroke that allows for performance preservation despite aging-related physiological changes, until catastrophic decline occurs [65]. The rapid deterioration post-breakpoint may relate to cumulative effects of reduced aerobic capacity and neuromuscular control [2,23].

Breaststroke: The late coincident breakpoint (82 years) combined with the most gradual post-breakpoint decline (1.02%/year) suggest that breaststroke may be the most resilient stroke to aging effects. Breaststroke’s biomechanical characteristics, including its longer glide phase, which provides recovery opportunities, and its reliance on timing and coordination rather than absolute power, may buffer against age-related physiological changes. The gradual decline suggests that older breaststroke swimmers retain functional capacity and efficiency longer than in other strokes [66].

Backstroke: The earliest performance breakpoint (47 years), with minimal post-breakpoint decline (0.30%/year), presents a unique pattern. The early decline onset may relate to backstroke-specific biomechanical demands: sustained neck extension and shoulder external rotation required for the backstroke pull challenge aging cervical and shoulder structures [67]. However, the slow subsequent decline suggests that backstroke swimmers who adapt their technique and training may stabilize performance after the initial age-related adjustments. The 25-year lag between performance and pacing breakpoints is particularly striking, suggesting that backstroke performance deterioration reflects primarily biomechanical or structural changes rather than pacing dysregulation [68].

Butterfly: The earlier pacing deterioration (67 years) compared to performance decline (72 years) indicates that butterfly swimmers experience loss of rhythm and coordination before measurable performance decline. Butterfly’s high technical demands and reliance on precise neuromuscular coordination make it particularly sensitive to age-related changes in motor control. The steeper post-breakpoint slopes for both pacing (3.52%/year) and performance (2.07%/year) suggest that butterfly represents the stroke most vulnerable to accelerated decline once aging effects accelerate [51].

Together, these patterns indicate that it is not only the age at which decline starts that matters, but also how quickly performance and pacing deteriorate thereafter, and that this tempo of decline is stroke-specific and mechanically determined.

### 4.7. Implications for Training and Performance Monitoring

Pacing variability generally begins to increase 30 years earlier than race time (age 52 vs. 82 years) and rises more severely after the breakpoint (2.82% vs. 0.51%/year). The relationship between the age breakpoint in pacing variability and performance changes also indicates that swimming strokes with a later breakpoint tend to have a greater post-breakpoint performance decrease. This suggests that a drop in pacing consistency may act as an early warning signal of performance decline and that an intervention strategy optimization for master swimmers requires precise targeting of critical age windows.

Beyond the age breakpoint, pacing variability generally increases more rapidly than overall race time. This sharper decline in pacing consistency could be indicative of the central nervous system effects of extreme aging. With age, the neural drive to the muscle, synaptic transmission velocity, and motor unit recruitment all decline, hampering the ability to maintain fast, repetitive movement patterns, even when the absolute velocity is relatively preserved [15,45,69].

Mechanistically, the earlier breakpoints in pacing variability compared with race performance are compatible with the hypothesis that aging-related changes in central nervous system function and neuromuscular control may impair the fine regulation of pace before substantially altering mean race speed [70]. In technically and energetically demanding strokes such as butterfly, small decrements in rhythm, timing and force production may quickly translate into difficulties maintaining a stable pace, especially under fatigue [34]. In strokes with more continuous propulsion or relatively more stable body positions, such as backstroke and breaststroke, pacing control may be preserved for longer or decline more gradually [71]. However, these mechanistic interpretations remain tentative, as the present analysis relied solely on race and split times and did not include direct measurements of stroke rate, biomechanical variables, muscle function or neural control.

Results of the study suggest that the development of an early warning system by real-time monitoring algorithms can detect initial signs of accelerated decline onset before reaching a critical age breakpoint across different swimming disciplines [72]. Age-specific intervention window calculation based on stroke specialization risk trajectories can help coaches define pre-breakpoint preparatory phases (10–15 years before breakdown), critical monitoring periods (±5 years), and post-breakpoint management strategies for evidence-based training adaptation protocols tailored to individual stroke characteristics, physiological aging patterns, and risk vulnerability profiles. The development of a risk scoring framework through a multi-factorial risk assessment combining acceleration rates, age breakpoints, and model reliability could help to quantify individual vulnerability to accelerated functional decline [73].

Personalized risk trajectory modeling for individual-level prediction models incorporating stroke specialization, performance history, and age-specific vulnerability patterns could be used for targeted intervention timing. The quantitative assessment of individual performance declines using model reliability indices across stroke specializations can provide a predictability method enabling evidence-based monitoring and early warning system calibration for timely aging detection.

The composite stroke-specific risk score was designed to integrate three dimensions of age-related decline onset (breakpoint age), speed (post-breakpoint slope) and model reliability (R^2^) into an intuitive descriptive framework [74]. By combining these elements, the score highlighted moderate vulnerability in freestyle, backstroke and butterfly, and lower risk in breaststroke. From a practical standpoint, this framework translates into distinct critical windows for heightened monitoring and intervention. While the risk score is not intended as an individual predictive tool, it offers coaches and clinicians a simple way to compare strokes and prioritize when stroke-specific preventive strategies may be most beneficial. Future studies should test whether similar scores derived from independent datasets have prognostic value for individual athletes.

### 4.8. Evidence from Exceptional Aging in Masters Athletes

The remarkable improvements observed in centenarian athletes following targeted training provide encouragement that age-related declines are not immutable. For example, a 100-year-old cyclist (Robert Marchand) improved maximal aerobic power by 13% following two years of cadence-focused training and set a new world record [21]. These findings suggest that neuromuscular and cardiorespiratory adaptations remain possible even at extreme ages. By analogy, stroke- or skill-specific interventions aimed at preserving rhythmicity, coordination and pacing stability may help maintain race competence in aging swimmers, particularly in strokes that appear more vulnerable to early pacing disruption [75,76].

### 4.9. Study Limitations

This study has several important limitations that should be considered when interpreting the findings.

Limited Mechanistic Data: The analysis was based exclusively on race and split times from official results. Direct measurements of stroke rate, biomechanical variables, neuromuscular measures, or physiological data (e.g., lactate thresholds, VO_2_max, electromyography) were not available. Consequently, any mechanistic interpretations regarding neuromuscular control, central nervous system function, or cadence remain speculative. The conclusions are therefore limited to behavioral manifestations of performance and pacing in competition and should not be extrapolated to underlying physiological mechanisms without additional validation studies.

Cross-Sectional Design and Selection Bias: The design was observational and essentially cross-sectional: performances from different swimmers, years, and age groups were analyzed together. Some swimmers likely contributed multiple results across events and championships. As a result, observations cannot be considered strictly independent, and cohort or selection effects cannot be ruled out. The study did not use mixed-effects models or track individual longitudinal trajectories, which would more precisely characterize within-athlete aging patterns and account for repeated measures from individual swimmers. Additionally, the analysis includes only swimmers competing at World Championships, representing a particular population of healthy and highly motivated older athletes. The timing and magnitude of the observed breakpoints may not be applicable to the broader masters swimming community, recreational swimmers, or those with chronic disease or disabilities.

Heterogeneity and Potential Confounding: In some analyses, performances from different race distances (100–800 m) and from both sexes were combined to maximize sample size. This may have masked sex-specific or distance-specific aging trends. For example, pacing strategies in 100 m sprints may differ substantially from 800 m distance races, and aging effects may be sex-specific. Future studies should examine these subgroups separately.

Effect Sizes and Explained Variance: Although pacing variability showed the strongest association with race time relative to other pacing indices, the effect sizes were small (r = 0.173), explaining only approximately 3% of the variance in performance. This suggests that while pacing is an important factor, other variables not examined in this study (e.g., aerobic capacity, metabolic efficiency, technique refinement, psychological factors, training history) also substantially influence performance. The composite stroke-specific risk score was derived from the segmented models and should be viewed as an exploratory descriptive tool rather than a validated predictive index. Future studies should seek to replicate these risk profiles in independent datasets and test their prognostic value at the individual level.

### 4.10. Deductions

Despite these limitations, the present findings provide data-driven information for long-term preparation and performance monitoring of master swimmers. First, they suggest that monitoring pacing variability across seasons and age groups may provide an earlier signal of accelerated decline than race time alone, potentially enabling preventive interventions 20–30 years before measurable performance deterioration. Second, they demonstrate the need for stroke-specific approaches to aging, for example, by reinforcing technical efficiency and neuromuscular robustness in butterfly as athletes approach their 60s and 70s or implementing earlier preventive strategies in backstroke to counteract the earlier onset of decline. Finally, the results suggest that maintaining a high-level of performance into very old age is not only a matter of sustaining aerobic capacity but also of preserving the ability to distribute effort optimally throughout the race. Consistent pacing yields better race times in competitive master swimmers [2,40,41].

## 5. Conclusions

The aim of this study was to identify a distinct age-associated breakpoint marking the onset of increased pacing variability and performance deterioration in master swimmers. Analysis of race and split-time data from World Aquatics Masters Championships revealed that pacing variability displays a measurable age-related breakpoint that occurs earlier than the breakpoint observed for overall race performance. The timing and magnitude of these breakpoints differed by sex, race distance and stroke.

Sex differences showed that women demonstrate a prolonged gap (~35 years) between pacing deterioration and performance decline in short-distance events, while men exhibit more synchronized decline trajectories across distances. This could suggest that neuromuscular precision for rapid effort distribution is particularly vulnerable to sex- and menopause-related changes, potentially reflecting protective effects of greater muscle mass preservation.

Freestyle and breaststroke showed coincident pacing and performance breakpoints (82 years), with freestyle demonstrating rapid post-breakpoint decline and breaststroke showing gradual deterioration. Backstroke displayed the earliest performance breakpoint (47 years) yet the slowest post-breakpoint rate of change, with pacing remaining stable until age 72 years. Butterfly combined early pacing deterioration (67 years) with subsequent performance decline (72 years).

These findings suggest that changes in pacing stability may precede observable declines in mean race speed in competitive master swimmers by decades, identifying pacing variability as an early, sensitive indicator of age-related functional decline. The sex, race distance and stroke-specific patterns revealed in this analysis provide coaches and clinicians with data-driven guidance on optimal timing and targets for preventive interventions. While these results are exploratory and require independent replication, they highlight pacing variability as a potentially informative descriptor of age-related performance changes. Longitudinal studies incorporating biomechanical and physiological measures are required to confirm these observations and to determine their practical relevance for training and performance monitoring in aging swimmers.

## Figures and Tables

**Table 1:** Participant characteristics by age group.

Age Group (Years)	Men (*n*)	Women (*n*)	Total (*n*)
95–99	2	3	5
90–94	36	14	50
85–89	88	53	141
80–84	196	176	372
75–79	284	272	556
70–74	403	387	790
65–69	588	564	1152
60–64	780	737	1517
55–59	760	709	1469
50–54	839	716	1555
45–49	713	657	1370
40–44	683	565	1248
35–39	578	415	993
30–34	771	559	1330
25–29	697	577	1274
Total	7417	6405	13,822

Distribution of participants across age groups and by sex. Age groups follow World Aquatics Masters Swimming classification (5-year intervals). Sample includes performers from 85 countries across seven continents.

**Table 2:** Descriptive variables by age and sex group.

Age Range and Sex	RT Mean	RT SD	CV Mean	CV SD	ST1 Mean	ST1 SD	ST2 Mean	ST2 SD	Δ Mean	Δ SD
M 25–29	115.1	10.3	8.2	3.3	91.0	4.6	105.3	4.0	−14.3	6.8
M 30–34	115.5	11.5	8.1	3.9	91.0	4.5	104.9	4.1	−13.9	6.5
M 35–39	115.7	10.2	8.2	3.5	90.8	4.5	105.2	4.3	−14.4	7.1
M 40–44	116.7	13.3	8.1	3.6	90.8	4.6	104.9	4.3	−14.1	7.1
M 45–49	117.8	14.5	7.6	3.5	91.2	4.4	104.5	4.2	−13.4	6.9
M 50–54	119.1	14.1	7.7	3.5	90.7	4.6	104.3	4.6	−13.6	7.2
M 55–59	119.6	15.0	7.6	3.7	90.9	4.7	104.1	4.5	−13.2	7.3
M 60–64	122.4	15.6	7.9	3.8	90.3	4.8	103.8	5.1	−13.4	7.7
M 65–69	125.8	19.0	7.6	3.9	90.8	4.8	103.4	6.5	−12.5	8.6
M 70–74	124.0	19.4	7.8	4.4	90.1	8.2	103.9	5.3	−13.8	10.5
M 75–79	129.8	20.9	7.6	4.1	90.5	9.2	101.9	10.3	−11.4	14.3
M 80–84	131.9	20.5	8.3	4.3	87.5	13.7	102.9	5.9	−15.5	15.0
M 85–89	125.8	28.4	8.7	4.4	89.9	5.1	103.9	7.7	−14.0	10.8
M 90–94	141.4	26.3	10.8	4.4	88.5	5.5	104.7	7.4	−16.3	10.0
M 95–99	122.2		5.2		96.3		103.7		−7.4	
F 25–29	117.4	7.9	7.3	3.0	91.9	3.3	104.4	3.7	−12.5	5.4
F 30–34	119.5	8.9	7.0	2.9	91.9	3.7	104.1	3.8	−12.2	5.6
F 35–39	119.8	9.6	7.0	3.1	91.7	3.8	104.1	4.4	−12.5	6.5
F 40–44	120.3	10.8	7.3	3.3	91.3	3.6	104.1	4.3	−12.8	6.1
F 45–49	123.7	12.0	7.2	3.1	91.6	3.7	103.8	4.3	−12.2	5.9
F 50–54	123.7	27.6	7.1	3.3	91.4	3.9	103.5	4.6	−12.2	6.3
F 55–59	129.8	16.1	7.1	3.3	91.4	3.9	103.2	4.8	−11.8	6.6
F 60–64	133.2	15.9	7.4	4.6	91.4	5.1	103.2	5.5	−11.8	8.2
F 65–69	135.0	18.6	7.8	5.4	90.8	8.3	103.9	7.4	−13.1	14.4
F 70–74	139.9	19.2	7.9	6.6	90.9	7.9	103.8	8.2	−12.9	14.1
F 75–79	135.4	17.4	8.3	8.1	90.6	10.2	103.8	10.5	−13.2	19.6
F 80–84	132.7	18.6	8.5	6.9	90.9	7.0	103.7	7.6	−12.8	12.0
F 85–89	142.2	28.9	7.2	3.1	91.4	4.5	103.3	4.3	−11.9	6.6
F 90–94	140.7	23.8	8.2	4.2	92.8	3.4	103.8	5.3	−11.1	7.0
F 95–99	191.7	40.5	9.1	4.0	92.0	7.2	102.2	6.8	−10.2	12.6

Mean values and standard deviations (SD) of Age ranges from 2529 to 95–99; M: male, F: female; RT: Race Time %WR (%); CV: pacing variability (%); ST1: Split time 1 half (%); ST2: Split time 2 half (%); Δ: Delta 1–2 half (%).

**Table 3:** Pacing variables as predictors of race performance: correlation analysis.

Predictor Variable	Correlation (r)	*p*-Value	95% CI
CV	0.173	<0.001	0.159–0.188
ST1	−0.147	<0.001	−0.162–−0.133
ST2	0.130	<0.001	0.115–0.144
Δ	−0.147	<0.001	−0.162–−0.133

Pearson correlation coefficients quantifying associations between pacing indices and overall race performance (Race Time %WR). CV: pacing variability (%); ST1: Split time 1 half (%); ST2: Split time 2 half (%); Δ: Delta 1–2 half (%).

**Table 4:** Segmented regression results: age breakpoints for race performance and pacing variability.

Parameter	Race Time %WR	Pacing Variability (CV)
Age Breakpoint (years)	82	52
95% CI for Breakpoint	80–84	50–54
Before Breakpoint		
Age Range	27–82	27–52
Annual % Change	+0.28	+0.18
R^2^	0.891	0.764
After Breakpoint		
Age Range	82–97	52–97
Annual % Change	+0.51	+2.82
R^2^	0.734	0.687
Acceleration Factor	1.82×	15.7×

Segmented linear regression results identifying age breakpoints at which the rate of change shifts for race performance and pacing variability. Race Time %WR is expressed as time relative to age- and sex group world record. CV is the coefficient of variation of within-race split durations. Annual % Change represents the estimated yearly increase in each outcome variable within each age segment. The pacing variability breakpoint (52 years) occurs 30 years earlier than the race performance breakpoint (82 years), and the post-breakpoint deterioration rate is 15.7 times steeper for pacing than for race performance, indicating that pacing instability is a sensitive early marker of age-related functional decline.

**Table 5:** Stroke-specific segmented regression results.

Stroke	BP RT (Years)	Post-BP RT Slope (%/yrs)	BP CV (Years)	Post-BP CV Slope (%/yrs)
Freestyle	82	6.58	82	3.15
Breaststroke	82	1.02	82	2.41
Backstroke	47	0.30	72	1.89
Butterfly	72	2.07	67	3.52

Stroke-specific segmented regression parameters. BP RT, age at breakpoint for race performance (Race Time %WR); post-BP slope RT, annual percentage change in race performance after breakpoint; BP CV, age at breakpoint for pacing variability (CV); post-BP CV slope, annual percentage change in CV after breakpoint.

**Table 6:** Stroke-specific composite risk scores and critical age windows.

Stroke	Composite Risk Score	Risk Category	Critical Age Window (Years)	Primary Concern
Freestyle	39.98	Moderate	80–89	Fast decline onset
Breaststroke	21.81	Low-Moderate	80–89	Gradual decline
Backstroke	31.52	Moderate	40–49	Early onset
Butterfly	34.70	Moderate	60–79	Pacing instability

Composite risk scores and descriptive categories for each swimming stroke. Scores integrate three dimensions: (i) age at performance breakpoint (40% weight), (ii) annual rate of post-breakpoint performance decline (40% weight), and (iii) model goodness-of-fit R^2^ (20% weight). Risk categories: Low (0–24), Low-Moderate (25–49), Moderate (50–74), High (≥75). Critical age windows indicate the decade(s) during which enhanced monitoring and preventive interventions may be most valuable for maintaining pacing stability and performance in each stroke.

## Data Availability

All data analyzed in support of the reported results can be found at the World Aquatics official website (https://www.worldaquatics.com).
